# Observational Learning and Generalization in Infant Grasps of Novel Tools

**DOI:** 10.1111/infa.70126

**Published:** 2026-09-11

**Authors:** Caroline Danforth, Jane Hirtle, Supreethi Sridhar, Amy Work Needham

**Affiliations:** ^1^ Department of Psychology and Human Development Peabody College of Education and Human Development Vanderbilt University Nashville Tennessee USA

## Abstract

Tool use is a critical skill acquired in infancy. Here, we tested whether infants could learn how to grasp a tool from observing an adult model and whether this learning generalized to a new task and to increasingly dissimilar tools. Infants between 13 and 19 months of age (*N* = 75; *M*_age_ = 16.31 months; SD_age_ = 1.73 months; 36 girls) observed an adult grasp a spatula‐shaped tool by the narrow end, wide end, or both ends in alternation to push pom‐poms through a tube during a modeling phase. Infants in a control condition observed an adult pour pom‐poms from one large container into another. During a test phase, infants were given an opportunity to use the same tool, a tool that differed only in color, and a tool that differed in both color and shape to activate one of two puzzle boxes. Each puzzle box required one specific grip of the tool for activation (e.g., gripping the narrow, handle end to insert the wide, flat end into a thin slot on the box; gripping the wide, flat end to insert the narrow, handle end into a small hole on the box). Before the adult demonstration, infants spent more time grasping the tool by the narrow end (akin to grasping a spoon by the handle rather than the scooping end). After the demonstration, infants used more wide end grasps for the appropriate puzzle box if they observed the adult grasp the tool by the wide end during modeling. Overall, infants were able to transfer their knowledge to the tool that differed in color, but they were less likely to activate their puzzle box with the tool that differed in both color and shape. Findings indicate that 13‐ to 19‐month‐old infants can learn a new use of a tool from observing an adult demonstration, but generalization is relatively narrow and limited.

## 1 Introduction

Effective use of hand‐held tools is an important achievement for young children because using tools expands the range of activities we can complete with our hands (Baber 2003). In most Western cultures, many activities of daily living involve tool use of some kind, whether in maintaining personal hygiene (using a toothbrush to clean teeth), preparing food (using utensils to cut and eat food), or writing (using a pen and paper to make a shopping list). Learning to use tools signals infants' growing coordination between cognitive and motor abilities, and it provides new affordances for effectively accomplishing goals in one's environment (Defeyter and German 2003; Kahrs and Lockman 2014). Despite its foundational significance, tool use remains an understudied area in developmental science, and there is still much to be learned about how children acquire, use, and refine these fundamental motor skills (Rachwani et al. 2020; Riede et al. 2021).

Infants' tool‐using skills are likely cultivated over time via two main processes: *observational learning* and *independent problem solving* (Berger and Adolph 2003; Chen et al. 1997; McCarty and Keen 2005; McGuigan and Whiten 2009; Rat‐Fischer et al. 2024; Want and Harris 2002). Observational learning is an important influence on young children's behavior in many situations (e.g., Bandura et al. 1961; Casler 2014; Want and Harris 2002), including when children are expected to use an implement in a certain way (Connolly and Dalgleish 1989). For instance, the knowledge of how to use a spoon to feed oneself may be transferred to children either through explicit demonstration by skilled tool‐users or through passive observation of others' tool use actions. Independent problem solving, on the other hand, occurs when infants face a challenge such as procuring a toy that is out of their reach, and they must determine whether a hand‐held tool can help them achieve their goal (Brown 1990; Chen et al. 2000; Nagell et al. 1993). Observational learning and problem solving need not work alone, however, and have been shown to work in concert with each other (Esseily et al. 2013; Rat‐Fischer et al. 2024).

Infants begin developing expectations about how tools are used based on their own observations well before they successfully use tools themselves. Studies with 6‐ to 9‐month‐old infants show that they generate expectations of an actor's goals based on their hand aperture during grasping (Daum et al. 2009). Thus, toward the end of the first year of life, when infants begin to use tools in accordance with their intended function (Barrett et al. 2007; Rodríguez et al. 2018), they likely have accumulated experience with common tools associated with daily activities. For example, infants who have experience either using or seeing others use spoons, which are typically grasped by the narrow, straight end, may develop expectations for how spoons and other similar tools are grasped. Indeed, shortly after infants typically begin combining objects and showing early examples of tool use in routine tasks (e.g., bringing food to the mouth with a spoon), they increasingly prefer to grasp the narrow end of a tool (in typically developing infants, this usually happens between 14 and 19 months of age; Kaur et al. 2020; McCarty et al. 1999).

Before infants can reliably carry out functional tool actions, they must first develop an understanding of how an object's shape, size, and perceptual features relate to the actions the object affords. Between 10 and 14 months of age, infants can acquire these abstract associations between objects and their affordances, and these are essential for engaging in functional, goal‐directed tool use (Horst et al. 2005; Keen 2011). Infants' early application of these new associations tends to be rigid, sometimes interfering with tool use. For example, Barrett et al. (2007) demonstrated that infants around 14 months of age tended to over‐generalize the use of a familiar object, such as a spoon, in a way that interfered with their ability to achieve a novel goal with the tool (e.g., activate a puzzle box by holding the round end of a spoon to insert the handle end into a small hole on the box). When given a novel, perceptually similar tool, however, these same infants were flexible in their application of the tool to achieve their goal (Barrett et al. 2007).

These findings raise new questions about which perceptual features guide infants' generalization of tool affordances to new contexts. Work on infant visual cognition shows that infants tend to focus more on differences in shape (e.g., form features) than on differences in color (e.g., surface features) (Needham 1999; Wilcox 1999). In the context of the current study, infants may be more likely to transfer what they have learned about one tool to another tool that differs only in color than to a tool that differs in both color and shape. Although previous work has shown that infants first demonstrate sensitivity to color differences in an object individuation context around 11.5 months (Wilcox et al. 2007), no studies have directly tested how this affects infants' ability to generalize knowledge about tools based on color and shape.

It is also important to consider the additional factors that influence infant learning more broadly. For example, violation of infants' expectations has been shown to be a particularly salient cue for learning (Stahl and Feigenson 2017). When infants observe an action or event that is not aligned with their previous experiences, they attend more closely and subsequently engage in more exploratory behavior (Perez and Feigenson 2022). In the context of tool use, young infants who have relatively limited experience with tools (e.g., primarily spoon use) may pay closer attention to the actions of a skilled social partner when they are using a tool in an unexpected way, such as grasping a spoon or spoon‐like tool by the wide, rounded end instead of the narrow, handle end.

### 1.1 Current Study

The current study aimed to examine how observing an adult model shapes infants' actions with a novel tool, and whether this learning generalizes to new tool‐using contexts. Specifically, we set out to (1) determine whether infants' behavior with a novel tool can be influenced (e.g., shifted away from a prepotent grasp strategy) by watching an adult's relatively brief demonstration with that tool in a different context; and (2) examine how differences in the features of tools (i.e., color and small shape changes) affect infants' transfer of what they learned about that new tool to increasingly dissimilar tools.

We tested these questions using both between‐ and within‐subjects manipulations. Between subjects, infants were randomly assigned to see one of four adult modeling demonstrations before being given one of two puzzle boxes to solve. These manipulations allowed us to test our hypothesis that even a brief opportunity for observational learning (i.e., watching an adult use a novel tool) would be sufficient to influence infants' subsequent actions with that tool. We expected infants to be more likely to solve their puzzle box when the adult's demonstrated actions matched the actions required to solve their puzzle box. Within subjects, all infants attempted to solve their puzzle box with each of three tools that varied based on different dimensions such as color, shape, and pattern. This aspect of our procedure allowed us to test our hypothesis that infants would be more successful in transferring their knowledge about the tool used in the modeling demonstration to another tool that differed in color only and less successful in transferring their knowledge to a tool that differed in both color and shape.

## 2 Method

### 2.1 Participants

The final sample included 75 infants between 13 and 19 months of age (*M*_age_ = 16.31 months; SD_age_ = 1.73 months; 36 girls). Caregiver reports indicated that 59 infants were White, 9 were Black, 2 were Asian, 3 identified as more than one race, and 2 caregivers did not report race. Regarding ethnicity, 4 infants were Hispanic or Latino, 66 were not Hispanic or Latino, and 5 caregivers did not report ethnicity. The data in the study were collected with approval from an institutional review board. Families were recruited from a large metropolitan area in which 48% of adults aged 25 years and older have completed at least a bachelor's degree, and the median household income is approximately $77,371. Recruitment was conducted through online advertisements, flyers placed in community centers, and phone calls to families who had previously agreed to be contacted about research opportunities.

Infants were randomly assigned to one of four modeling conditions (described in detail below): Narrow end grip (*n* = 16; 8 girls; *M*_age_ = 16.1; SD_age_ = 1.6), wide end grip (*n* = 17; 9 girls; *M*_age_ = 16.5; SD_age_ = 1.5), both grips (*n* = 20; 10 girls; *M*_age_ = 16.6; SD_age_ = 1.8), and non‐tool grip control (*n* = 22; 9 girls; *M*_age_ = 16.1; SD_age_ = 2.0). An a priori power analysis was not conducted for this study. The available sample included at least 16 infants per group, and all 75 were included in the primary analysis of probability of success. Data from an additional 22 infants were collected and excluded due to significant developmental delay (*n* = 2), fussiness (*n* = 3), experimental error (*n* = 8), and infants solving the task at baseline (*n* = 3 used finger to activate light box; *n* = 3 used tool to activate light box; *n* = 3 used tool to activate sound box).

### 2.2 Procedure and Materials

**FIGURE 1:**
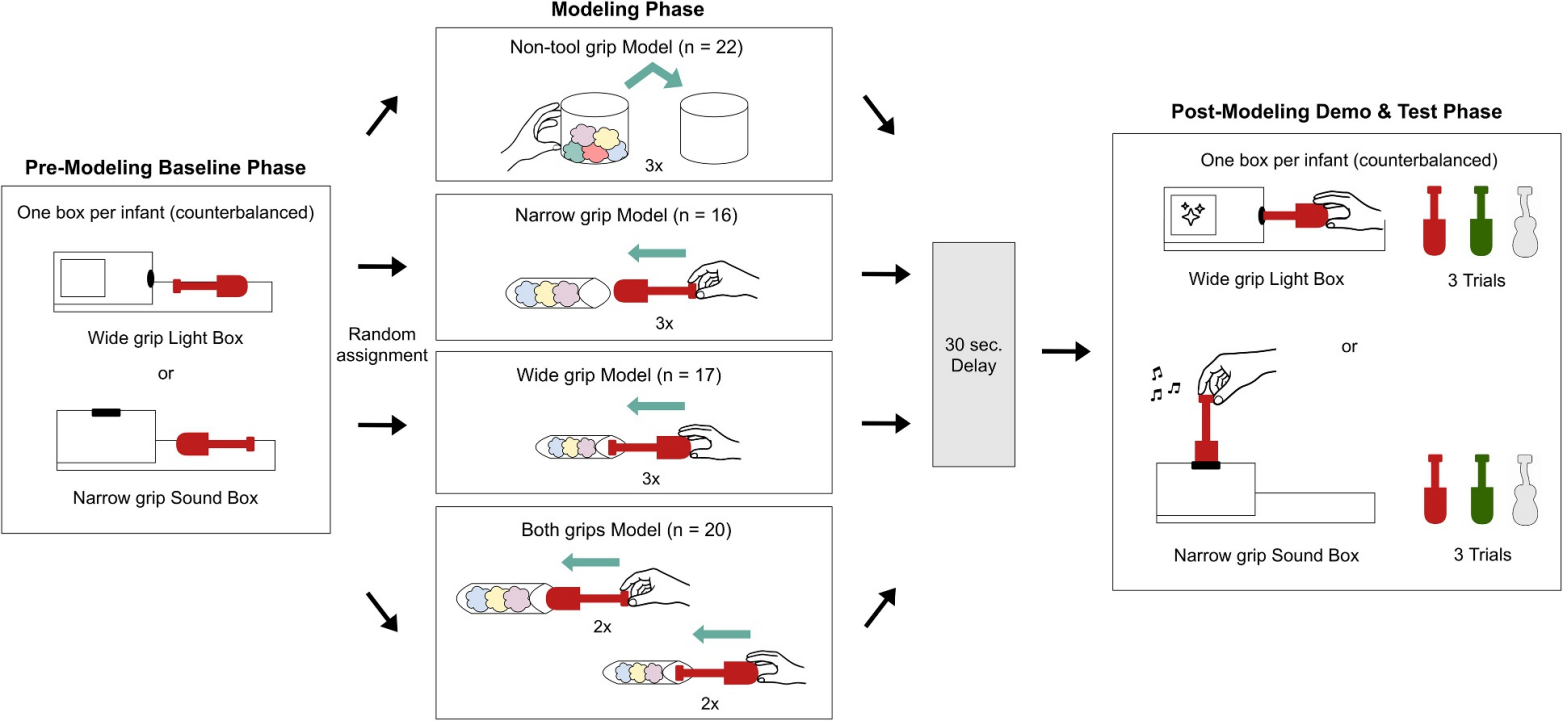
Experimental design. All infants completed three phases during the study. Pre‐Modeling Baseline: Infants had an opportunity to explore the red tool and a puzzle box. Modeling: Infants observed an adult carrying out an action with the red tool. Post‐Modeling Demo & Test: An adult demonstrated how to use the red tool to solve the puzzle box, and infants had three opportunities (each with a different tool) to solve it.

Overview: Infants were seated on their caregiver's lap at a table across from the experimenter during testing. Caregivers were instructed to refrain from providing any instruction or guidance to the infant, and four video cameras recorded the experiment from unique angles (directly above, to the infant's immediate left and right, and head‐on) for later data coding. The experiment followed the same sequence for all infants, consisting of a Pre‐Modeling Baseline Phase, a Modeling Phase, and a Post‐Modeling Demo and Test Phase (see Figure 1).

**FIGURE 2:**
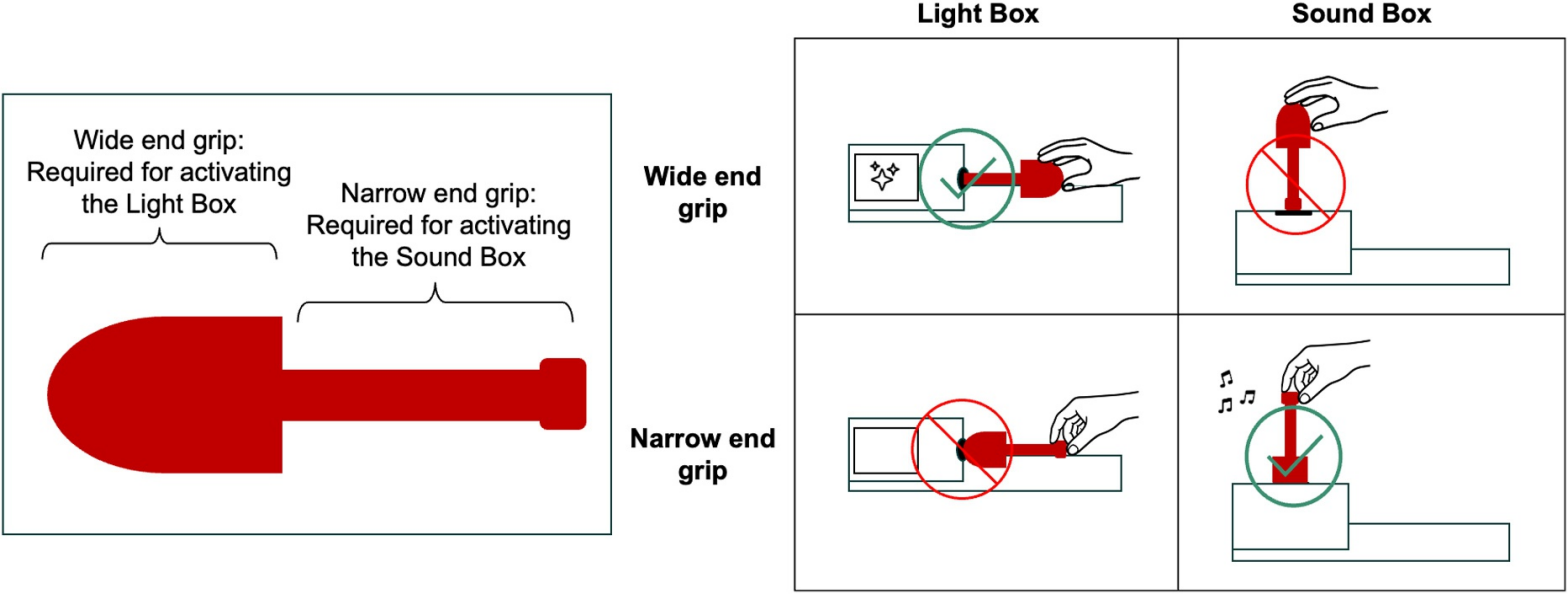
Puzzle box activation. The tool used during the baseline phase had a wide, flat end and a narrow, cylindrical end. To activate the light box, infants needed to grip the tool by the wide end to insert the narrow end into a small hole on the side of the box. To activate the sound box, infants needed to grip the tool by the narrow end to insert the wide end into a slit on the top of the box. Attempting to insert the wide end of the tool into the light box or the narrow end of the tool into the sound box would not result in activation.

Pre‐modeling baseline phase: The purpose of this phase was to ensure that infants would not spontaneously solve either of the puzzle boxes before observing an adult model. Infants who solved their puzzle box (*n* = 9) continued the study, but they were only included in analyses assessing their grip of the tool during baseline. The custom‐made puzzle boxes were a light box measuring 12.5 (W) × 15 (L) × 12 cm (H), and a sound box measuring 12.5 × 14 × 13 cm. Inserting a narrow object through a small circular hole (2 cm diameter) on the right side of the light box activated an infrared sensor that illuminated green and red LED lights visible through a front‐facing Plexiglas window. Inserting a thin, flat object into a narrow slit (4.5 × 1 cm) on the top of the sound box activated an infrared sensor that triggered an electronic tone. During this phase, the experimenter placed a red tool, which was approximately 16.25 cm long with a wide, flat end (0.5 × 7.5 cm) connected to a narrow cylindrical shaft (0.75 cm diameter) ending in a slightly larger knob (1.25 cm diameter) (see Figure 2, left panel) on the table next to the puzzle box. The tool's positioning relative to the puzzle box (e.g., narrow end facing the box vs. wide end facing the box) was counterbalanced between infants. The light box could only be solved by grasping the tool by the wide end and inserting the narrow end into the hole. The sound box could only be solved by grasping the tool by the narrow end and inserting the wide, flat end into the slit on the top of the box (see Figure 2, right panel). Infants were permitted 30 seconds of exploration with each puzzle box and the red tool for a total of two trials.

Modeling phase: Infants viewed the experimenter model one of four actions with pom‐poms (summarized in Table 1). Three actions involved the red tool, and one action did not involve any tools. Although infants' attention to the modeling demonstration was not directly measured here, experimenters made every effort to attract infants' attention at the beginning of the trial and waited for them to look in their direction before modeling the action.

**TABLE 1:** Summary of modeling conditions.

Model	Tube	Pom‐poms	Modeled grip of tool	Total demos
Non‐tool grip	—	Large and small	No tool used; pom‐poms poured between clear plastic bins	3
Narrow end grip	4.5 × 11 cm (large tube)	Large only	Narrow end grasped; wide end pushed pom‐poms out	3
Wide end grip	2.5 × 9 cm (small tube)	Small only	Wide end grasped; narrow end pushed pom‐poms out	3
Both grips	Large and small tube	Large and small	Narrow end grasped 2x; wide end grasped 2x	4

*Note:* The experimenter demonstrated one of four actions during the modeling phase. The non‐tool grip model consisted of pouring pom‐poms from one large plastic bin into another. The narrow end grip and wide end grip conditions consisted of gripping one end of the red tool and using the other end to push the pom‐poms out of a tube. Infants in the both grips condition observed both grip demonstrations. Tube dimensions are reported W × L.

Non‐tool grip: The experimenter placed two clear plastic bins side by side on the table, one filled with pom‐poms and one empty (see Figure 1, Non‐tool grip Model) and poured pom‐poms from one bin into the other before replacing the bin in its original position. This action was demonstrated 3 times.

Narrow end grip: The experimenter placed a large, clear plastic tube filled with large pom‐poms (4.5 cm in diameter) on the table beyond the infant's reach. She then grasped the narrow end of the red tool and used the wide end of the tool to push the pom‐poms out of the tube (see Figure 1, Narrow grip Model). This action was demonstrated 3 times.

Wide end grip: The experimenter placed a narrow, clear plastic tube filled with small pom‐poms (2.5 cm in diameter) on the table beyond the infant's reach. She then grasped the wide end of the red tool and used the narrow end of the tool to push the pom‐poms out of the tube (see Figure 1, Wide grip Model). This action was demonstrated 3 times.

Both grips: The experimenter gave two demonstrations of each tool grip type using the red tool (see Figure 1, Both grips Model). The order of demonstration was counterbalanced across infants.

Post‐modeling demo and test phase: After a 30‐s delay, all infants were given two opportunities to observe the experimenter activate their assigned puzzle box before the first test trial only. Infants were then given three 30‐s test trials to solve their puzzle box, each with a different tool (see Figure 1, Post‐Modeling Demo & Test Phase). For the first test trial, infants were given the same tool used during modeling and demonstrations (red tool). For the second and third test trials, infants were given a tool that differed only in color (green tool) followed by a tool that differed in color, shape, and pattern (white tool). The white tool had a violin‐shaped flat end and a curved shaft. Each tool's wide end could fit into the slit of the sound box, but not the hole on the light box. The narrow end of each tool could fit into the hole in the light box, but not the slit on the sound box.

Hand preference task: All infants completed a brief, 3‐trial handedness assessment where the experimenter offered a small plastic toy to the infant and recorded the hand (or hands) the child used to grasp the toy. Infants' responses were recorded in real time by the experimenter during the experiment.

### 2.3 Measures

Overview: A trained coder annotated all performance measures (baseline grip type, success, and latency to success) from the recorded videos of each infant. The original plan for the study was for each infant to receive counterbalanced blocks of test trials with the light box and the sound box. Preliminary inspection of the data made clear that infants did not maintain enough interest in the testing materials to complete both blocks of test trials. Therefore, only the first block of test trials was analyzed, effectively making the test box (light box or sound box) a between‐subjects variable.

Baseline grip type: The location of the infant's grip on the tool (narrow end, round end, middle, or both ends with both hands) during the baseline phase was determined. For each infant, the proportion of time spent in each grip location was calculated from their overall grip duration. To ensure inter‐observer reliability, a reliability coder classified grip location in 25% of each 30‐s baseline (three 2.5‐s segments randomly selected from the beginning (first 10 seconds), middle (second 10 seconds), and end (third 10 seconds) of each video. Reliability between coders was calculated using Cohen's kappa formula and was found to be high for grip type, *κ* = 0.93.

Success: Success on the light box was defined as inserting the narrow end of the tool into the hole of the light box and activating the box at least once during a trial. Success on the sound box was defined as inserting the wide end of the tool into the slot of the sound box and activating the box at least once during the trial. Throughout this paper, we also use the terms “activate” and “solve” to indicate success on the puzzle boxes. Success was coded as a binary variable indicating whether infants solved the puzzle box at any point during the trial, and it was recorded live by the experimenter. With three test trials, there was a maximum of three success observations per infant. A second trained coder coded the full dataset (one trial observation for one participant was excluded due to corrupted video) to ensure reliability of the success variable. Reliability between coders was calculated using Cohen's kappa formula, and was found to be high for success, *κ* = 0.97. The instances of disagreement were assessed and resolved, and the main coder's annotations were retained in the final dataset.

Latency to success: For all successful trials, a main coder determined the time that elapsed from the presentation of the puzzle box to the activation. A second trained coder coded a random subset (*n* = 34) of successful trials for reliability. Because latency is a continuous variable, the intraclass correlation coefficient (ICC) for double‐coded trials was calculated and found to be high, ICC = 0.995.

## 3 Results

### 3.1 Baseline Grip Preference

**FIGURE 3:**
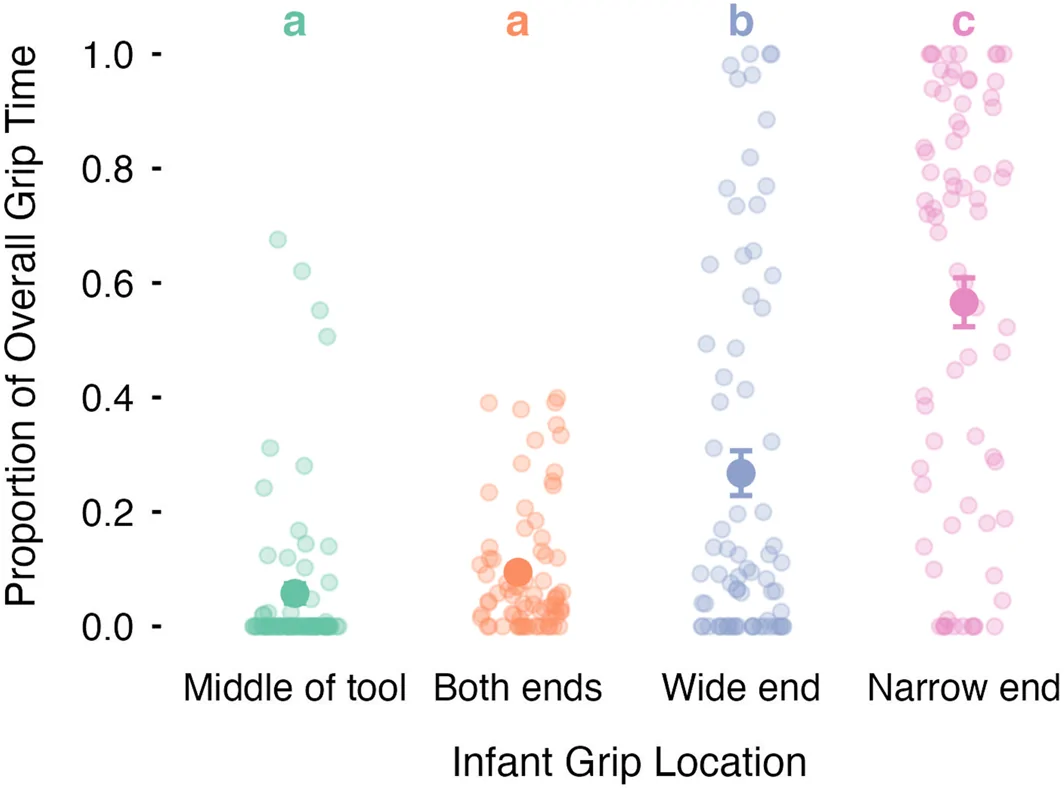
Infant grip location during baseline. The proportion of time infants spent using each grip type during the baseline phase. Each individual dot within a grip type represents a single infant, the large solid dot represents the mean, and error bars reflect standard error. On average, infants spent more time grasping the tool by the narrow end compared to all other grip types.

A 4 (grip type) × 2 (puzzle box) repeated‐measures ANOVA was conducted on the proportion of the baseline phase infants spent using each grip type. Grip type was treated as a within‐subjects factor and puzzle box as a between‐subjects factor. Two infants were excluded from this analysis because their proportion of missing data was more than 2 SDs above the mean. We found that infants grasped the narrow end most frequently (*M* = 0.57, SD = 0.36), *F* (3, 213) = 42.93, *p* < 0.001, *ηp* ^*2*^ = 0.377 (see Figure 3). Follow‐up pairwise comparisons with Bonferroni correction (*m* = 6) confirmed that this grip type occurred more frequently than a grip of the wide end (*M* = 0.27, SD = 0.33), *p* = 0.002, a two‐handed grip on either end of the tool (*M* = 0.10, SD = 0.12), *p* < 0.001, and the middle of the tool (*M* = 0.06, SD = 0.15), *p* < 0.001. There was no overall effect of puzzle box, *F* (1, 71) = 0.92, *p* = 0.34, *ηp* ^*2*^ = 0.013, and no significant interaction between grip type and puzzle box, *F* (3, 213) = 1.16, *p* = 0.33, *ηp* ^*2*^ = 0.016. In sum, before infants were given any indication of how to grasp the tool, they spent most of their baseline phase gripping the tool by the narrow end, and this was true for infants regardless of the puzzle box they were assigned.

### 3.2 GEE Modeling of Success and Latency to Success

Generalized estimating equation (GEE) modeling was used to analyze both success and latency outcomes. A GEE model was used for the primary analysis instead of an ANOVA here due to the binary nature of the main outcome variable (success) and the clustered nature of the data (3 observations per infant), as it handles correlated observations well. GEE modeling has often been used in studies with designs similar to the current study (Barrett et al. 2007; Kretch et al. 2014; Osina et al. 2013). Infants who solved the task at baseline (*n* = 3 used finger to activate light box; *n* = 3 used tool to activate light box; *n* = 3 used tool to activate sound box) were not included in these models.

### 3.3 Model 1: Probability of Trial Success

Model characteristics: In the final GEE model predicting success with a logit link (chosen due to its appropriateness for a binary outcome variable such as success/failure) and an exchangeable correlation structure, four predictor variables were included. Throughout the binary GEE analyses, *b* represents the unstandardized regression coefficient in log‐odds. Odds ratios (OR) are reported throughout the binary GEE results to facilitate interpretation.

The first three variables were between‐subjects: *modeling group* (non‐tool grip control, narrow grip, wide grip, both grips), *puzzle box* (light box, sound box), and *age* (continuous). The fourth variable, *tool type* (red, green, white), which corresponded to the first, second, and third trials, respectively, was within‐subjects. Sex and handedness were initially included as potential predictors, but neither variable significantly influenced outcomes, and they were therefore excluded from the final model.

The model also included an interaction between task and condition. Because we were primarily interested in how performance varied on the light box task in response to different modeling types, we specified the non‐tool grip control condition, the first trial, and the light box task as the reference category. The resulting model revealed how each experimental condition and each subsequent trial compared to this group. We were primarily interested in performance on the light box task because it required a grasp that was likely not the most habitual one for infants at this age (i.e., a wide‐end grasp was required for success), whereas the sound box required a familiar narrow‐end grasp (Barrett et al. 2007). Thus, we expected that the infants' previous experience grasping the narrow end of tools, coupled with the design of these tasks, would result in varying success rates on the puzzle boxes. Raw performance metrics on the test trials that informed the GEE model are reported below (see Table 2).

**TABLE 2:** Proportion of infant successes by condition and tool type.

		Modeled grip			
Puzzle box	Tool	Non‐tool	Narrow end	Wide end	Both
Light box	Red	4 (33.3%)	2 (22.2%)	7 (77.8%)	5 (62.5%)
Light box	Green	4 (33.3%)	2 (22.2%)	7 (77.8%)	5 (62.5%)
Light box	White	4 (33.3%)	2 (22.2%)	5 (55.6%)	4 (50.0%)
Sound box	Red	7 (70.0%)	6 (75.0%)	5 (71.4%)	8 (66.7%)
Sound box	Green	6 (60.0%)	4 (50.0%)	4 (57.1%)	7 (58.3%)
Sound box	White	4 (40.0%)	3 (37.5%)	4 (57.1%)	6 (50.0%)

*Note:* This table shows raw performance metrics (number of successful infants (proportion)), not the probabilities of success obtained by the GEE model. The light box required a grip of the wide end of the tool for success. The sound box required a grip of the narrow end of the tool.

Age: The chances of success on both tasks overall increased as a function of age. Older infants were more likely to solve the tasks than younger infants, *b* = 0.673, SE = 0.15, Wald *χ* ^*2*^ (1) = 20.53, *p* < 0.001. For each additional month of age, the log‐odds of successfully solving the task increased by 0.673, which corresponds to roughly doubling the odds of success (OR = 1.96).

Puzzle box: There was no overall effect of puzzle box, *b* = 0.78, SE = 0.89, Wald *χ* ^*2*^ (1) = 0.77, *p* = 0.380, OR = 2.18. The predicted probability of success for infants assigned to the light box was 42.9% (SE = 0.09, 95% CI [0.27, 0.60]), while the predicted probability of success for infants assigned to the sound box was 60% (SE = 0.09, 95% CI [0.42, 0.76]). Although the estimated odds of success were 2.18 times greater for infants assigned to the sound box compared to the light box, the difference was not statistically significant. In other words, infants were not more or less likely to solve one puzzle box over the other.

**FIGURE 4:**
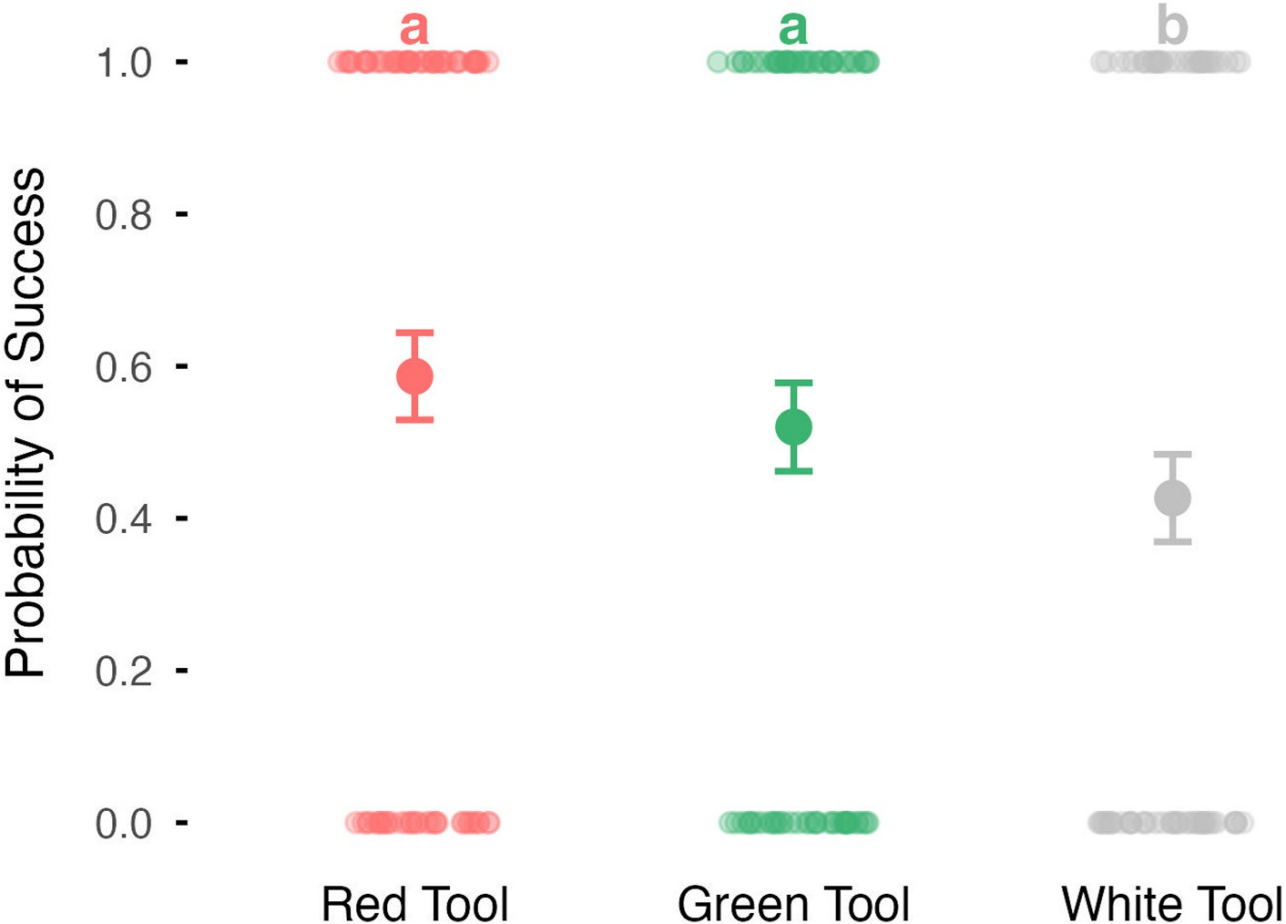
Probability of success by tool. The probability of success during test while using the red tool (used during modeling), green tool (same shape as red tool, different color), and white tool (different shape, different color). Large dots represent the estimated probability of success with each tool, and error bars reflect standard error.

Tool type: Collapsed across modeling conditions, there was no significant difference in the predicted probability of success between the red tool (*M* = 0.62, SE = 0.07, 95% CI [0.48, 0.74]) and the green tool (*M* = 0.53, SE = 0.08, 95% CI [0.38, 0.67]), *b* = −0.38, SE = 0.23, Wald *χ* ^*2*^ (1) = 2.83, *p* = 0.092, OR = 0.68 (see Figure 4). When using the white tool (*M* = 0.40, SE = 0.07, 95% CI [0.27, 0.54]), infants had less than half the odds of successfully solving the task compared to using the red tool, *b* = −0.90, SE = 0.23, Wald *χ* ^*2*^ (1) = 15.72, *p* < 0.001, OR = 0.41. Infants generalized their knowledge about the red tool's function to the green tool, which differed in color only, but not to the white tool, which differed in color, shape, and pattern pointing to a limitation in these infants' ability to generalize function across more than one perceptual dimension.

**FIGURE 5:**
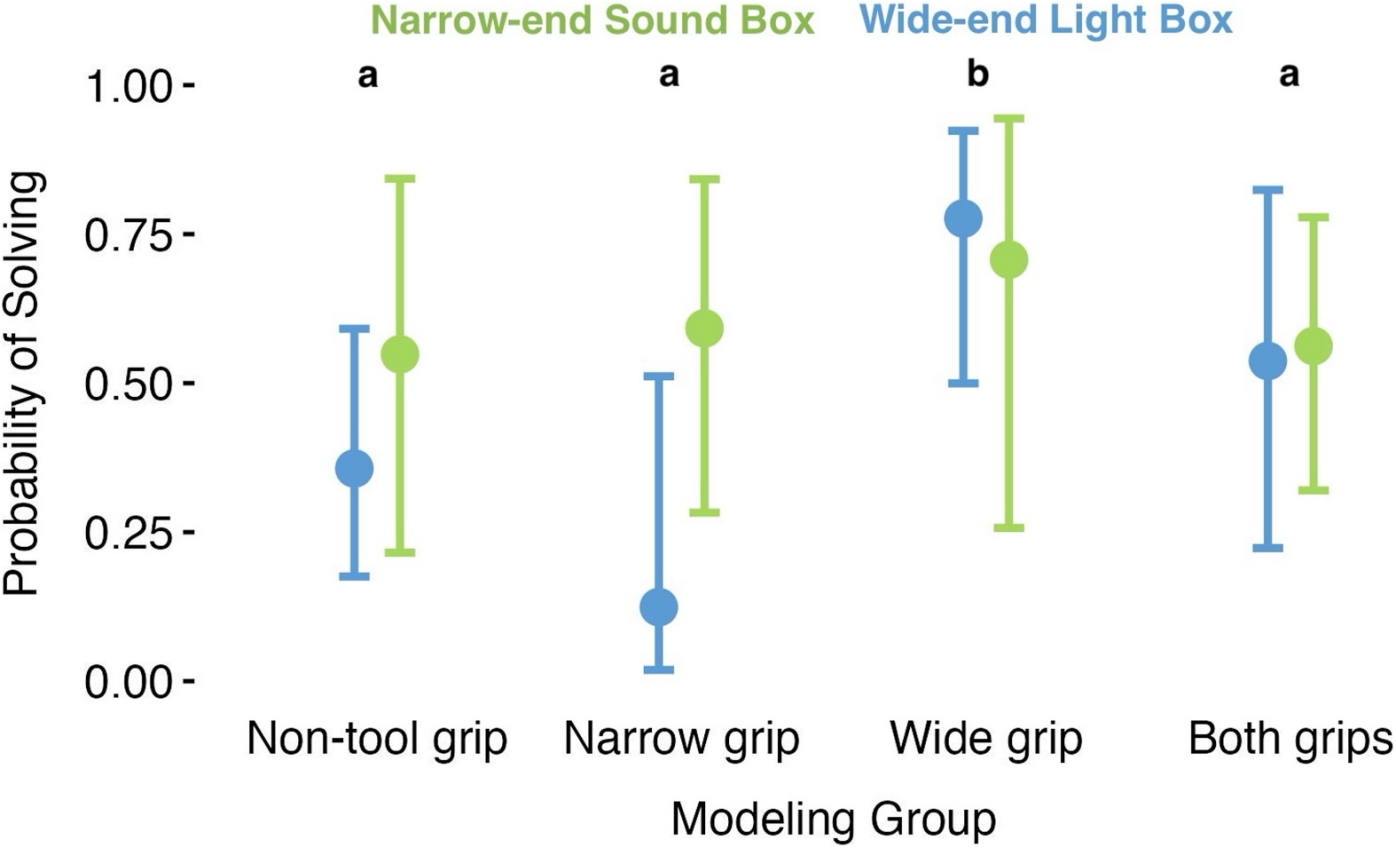
Probability of solving each task by modeling group. The probability of solving the puzzle boxes during test separated by which modeling infants observed prior to test. Large dots represent the predicted probability of success for each puzzle box (green: sound box; blue: light box). Error bars reflect standard error. Black letters indicate group‐level differences: Infants who observed a wide grip model were more likely to solve their puzzle box compared to all other modeling conditions.

Modeling group: The type of modeling infants observed significantly influenced their probability of success (see Figure 5). Infants who observed a wide grip model were more successful (*M* = 0.78, SE = 0.11, 95% CI [0.50, 0.92]) than infants who observed a non‐tool grip model (*M* = 0.12, SE = 0.11, 95% CI [0.02, 0.51]), *b* = 1.83, SE = 0.79, *Wald χ* ^*2*^ (1) = 5.39, *p* = 0.020, OR = 6.25. The predicted probability of success for the narrow grip modeling group was not significantly different from the non‐tool grip group (*M* = 0.36, SE = 0.11, 95% CI [0.18, 0.59]), *b* = −1.36, SE = 1.13, *Wald χ* ^*2*^ (1) = 1.47, *p* = 0.226, OR = 0.26. Infants exposed to both grips during modeling were not more or less likely to activate their puzzle box compared to those who viewed a non‐tool grip model (*M* = 0.54, SE = 0.18, 95% CI [0.22, 0.82]), *b* = 0.74, *p* = 0.394, OR = 2.10. There were no significant interactions between modeling type and puzzle box. For both puzzle boxes, infants who observed a wide grip model performed better overall than infants who observed a non‐tool grip. Infants who observed a narrow grip and those who observed both grips performed similarly to the non‐tool grip group.

### 3.4 Model 2: Latency to Solve

Model characteristics: In the final GEE model predicting latency to success with an identity link (chosen due to its appropriateness for a continuous outcome variable such as time) and an exchangeable correlation structure, five predictor variables were included. Trials where infants timed out at 30 seconds were not analyzed; only trials where infants solved their puzzle box were included in analysis. The first four variables were between‐subjects: *modeling group, puzzle box, age*, and *sex* (male, female). The fifth variable, tool type, was within‐subjects. The outcome variable of interest for this model (the number of seconds it took infants to solve their puzzle box) was a positively skewed variable, so we log‐transformed it prior to analysis to meet assumptions of normality. To facilitate interpretation, estimated regression coefficients are converted to percentage differences in latency on the original scale throughout the continuous GEE results. The model also included an interaction between task and condition, and as in the model for trial success, we specified the non‐tool grip control condition, the first trial, and the light box task as the reference category to reveal how each experimental condition and each subsequent trial compared to this group.

Sex and age: Male infants solved their puzzle box significantly faster than female infants, *b* = −0.270, SE = 0.12, Wald *χ* ^*2*^ (1) = 5.28, *p* = 0.021. Specifically, male infants demonstrated a 0.270‐unit lower log‐transformed latency than female infants, which corresponds to approximately 24% shorter solution times on average (e^−0.270^ = 0.763). The latency to solve both puzzle boxes overall decreased as a function of age. Older infants solved their puzzle box faster than younger infants, *b* = −0.106, SE = 0.04, Wald *χ* ^*2*^ (1) = 5.99, *p* = 0.014. Specifically, older infants demonstrated a 0.106‐unit lower log‐transformed latency than younger infants, corresponding to approximately 10% shorter solution times on average (e^−0.106^ = 0.899).

Puzzle box: There was an overall effect of puzzle box, *b* = 0.63, SE = 0.15, Wald *χ* ^*2*^ (1) = 18.97, *p* < 0.001, such that infants assigned to the light box (*M* = 4.83s, SE = 0.31) solved their puzzle box faster than infants assigned to the sound box (*M* = 6.96s, SE = 0.51). In the model, the sound box was associated with a 0.63‐unit increase in log‐transformed latency values relative to the light box, or approximately 88% longer solution times on average (e^0.63^ = 1.878).

Tool type: Collapsed across modeling conditions, there was no significant difference in the time it took infants to solve their puzzle box with the red tool (*M* = 6.36s, SE = 0.61) compared to the green tool (*M* = 5.65s, SE = 0.52), *b* = −0.12, SE = 0.14, Wald *χ* ^*2*^ (1) = 0.70, *p* = 0.404, or white tool (*M* = 5.43s, SE = 0.48), *b* = −0.16, SE = 0.14, Wald *χ* ^*2*^ (1) = 1.23, *p* = 0.267.

**FIGURE 6:**
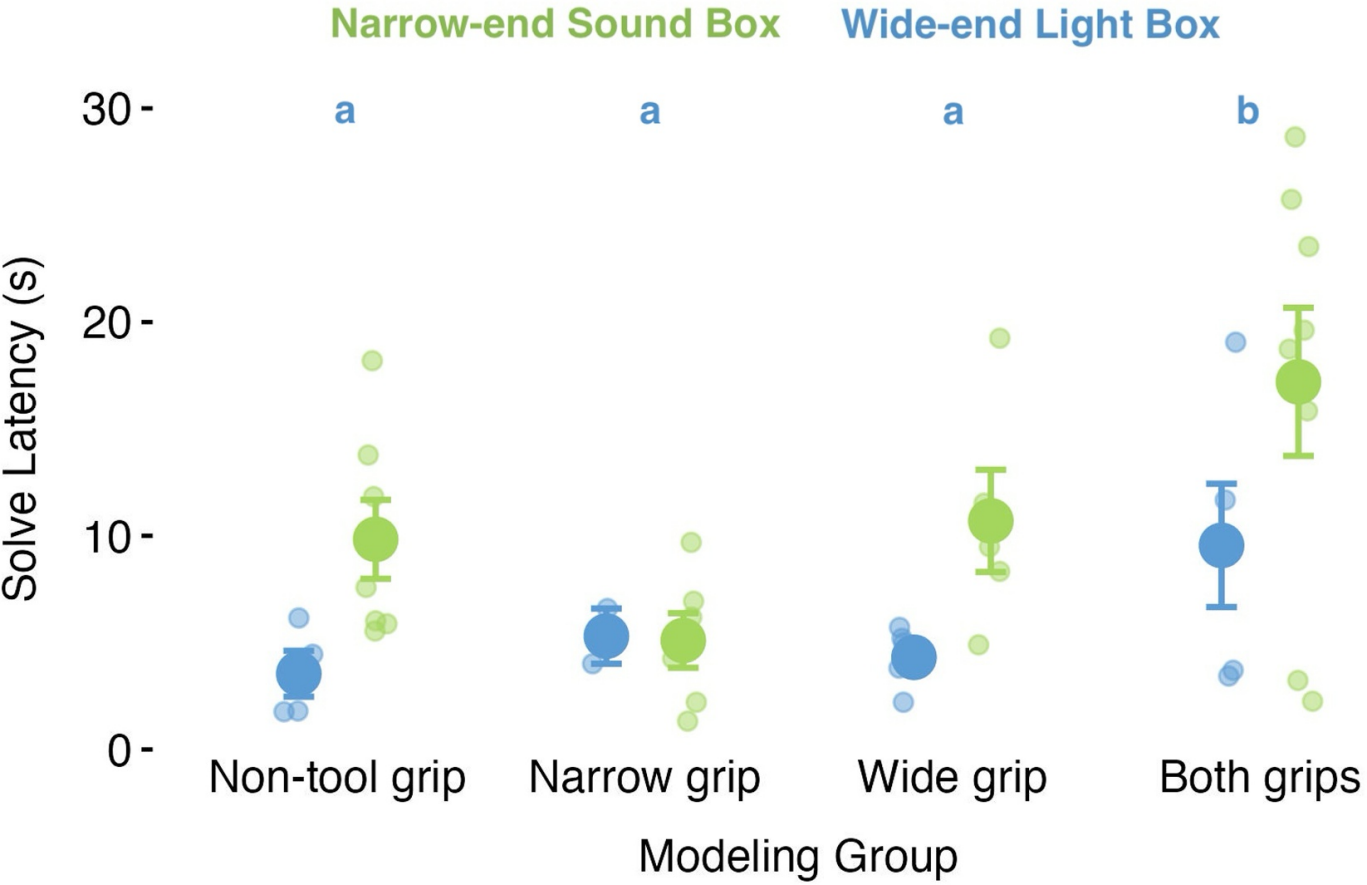
Latency to success with the red tool by modeling group. Latency values are represented for success on the first trial only with the red modeling tool. Non‐successful attempts that timed out at 30 seconds were not included in this analysis and are not reflected here. Large dots represent average latencies to success on each puzzle box (green: sound box; blue: light box), and small dots represent latency values for individual infants. Error bars reflect standard error. Blue letters represent group‐level differences for the light box task only: Infants who observed both grips took longer to solve the light box than infants in all other modeling conditions.

Modeling group: The time it took infants to solve their puzzle box when they were in the narrow grip modeling group (*M* = 4.37s, SE = 0.50) did not differ from infants in the non‐tool grip control condition (*M* = 5.96s, SE = 0.49), *b* = −0.20, SE = 0.13, Wald *χ* ^*2*^ (1) = 2.38, *p* = 0.123 (see Figure 6). Latency values for the wide grip modeling group (*M* = 6.17s, SE = 0.75) were also similar to the non‐tool grip modeling group, *b* = 0.11, SE = 0.23, Wald *χ* ^*2*^ (1) = 0.21, *p* = 0.644. Infants exposed to both grip modeling (*M* = 7.03s, SE = 0.57) took significantly longer to solve their puzzle box compared to infants in the non‐tool grip condition, *b* = 0.52, SE = 0.14, Wald *χ* ^*2*^ (1) = 14.82, *p* < 0.001. Specifically, the model estimates about 68% longer solve times for the both grips modeling condition compared to the non‐tool grip control (e^0.52^ = 1.68). This main effect of modeling group was modified by an interaction with puzzle box, *b* = −0.71, SE = 0.22, Wald *χ* ^*2*^ (1) = 10.84, *p* < 0.001. Further investigation into this interaction revealed that this effect of both grips modeling was only true for the light box, which required a grasp on the wide end of the tool, *t* (101) = 3.85, *p* = 0.001. In other words, infants who observed both grips modeled before the light box took longer to solve their puzzle than infants who viewed non‐tool grip modeling. There were no effects of modeling group on sound box performance.

## 4 Discussion

In the present study, we employed a lab‐based experiment to investigate the role of observational learning and generalization in infants' tool‐using behaviors. Infants were provided with an opportunity to see an adult either a) accomplish an action with no tool, b) grasp a novel tool on one end or the other to perform a goal‐directed action, or c) use the tool to perform the goal‐directed action by gripping one end or the other on alternate trials (seeing two different grips of the same tool). Next, infants were given a chance to use the same tool and increasingly dissimilar tools to complete a different goal‐directed action. Results showed that, before observing any adults' actions with the tool, infants preferred to grasp the tool by the narrow end, indicating an a priori preference. We also found that infants' grasp of the tool after observing an adult model was influenced by their observation of the adult's use of the tool. Finally, infants were more likely to extend their knowledge about the modeled tool to one that differed only in color than to one that differed in both shape and color.

Our findings provide clear evidence that infants' use of a novel tool was influenced by the brief actions they observed earlier in the study. Even though all infants saw a demonstration of their puzzle box solution at the beginning of their test trials, they still performed differently depending on the tool behavior they observed during the modeling phase of the study. This was the case even though the test task infants were given (solving a puzzle box) was different from the task they saw the adult engaged in during modeling (pushing pom‐poms out of a tube). We discuss these findings and their implications in more detail below.

### 4.1 Observing a *Brief* Model Influences Infants' Subsequent Tool Use

We report here clear evidence that the brief tool actions infants saw during the modeling phase influenced how they grasped and used the tools during the test phase. For example, infants who viewed a wide grip model were more likely to activate their puzzle box compared to those who observed a non‐tool grip action. If infants do have an existing expectation that the narrow end of a tool will be grasped, a concept that is consistent with our finding that infants spent more time grasping the tool by the narrow end during the baseline phase, then the experimenter's grasp of the wide end of the tool during modeling would have been unexpected. This could have led to increases in their attention to their own grasping behavior at test and to whether their grip was appropriate for the goal outcome.

As the findings from Feigenson and colleagues mentioned earlier (Perez and Feigenson 2022; Stahl and Feigenson 2017), observing unexpected events leads to increases in infants' visual and manual exploration of objects involved in those events. Thus, this experience may have disrupted an existing, habitual response pattern (e.g., grasping by the narrow end) and facilitated more deliberate, task‐specific motor planning. A similar result was obtained in a recent study from our lab that involved different tools and design: an experience in the study that violated infants' expectations may have prompted them to attend more closely to their subsequent actions with the tools (Danforth et al. manuscript in preparation).

While we anticipated that the rates of success would vary as a function of both modeling condition and puzzle box, we found no evidence for this predicted interaction. Descriptively, success rates appear to differ across puzzle boxes within some modeling conditions. For example, among infants in the non‐tool control condition, 33% successfully activated the light box, while 70% activated the sound box. This apparent difference was not supported by the GEE model, however, as it revealed no significant interaction between modeling condition and puzzle box. Instead, our results suggest that modeling effects, where they were significant, held true for both puzzle boxes (e.g., infants who viewed both wide *and* narrow grip models were just as likely to succeed as those who observed a non‐tool grip model, regardless of puzzle box). Nevertheless, the tendency toward greater success on the sound box was unsurprising. All infants were given an opportunity to observe an adult demonstrate the solution to their puzzle box immediately before their test trials, and the sound box required a familiar narrow grip of the tool that infants already tended to use (see Figure 3).

We did find, however, that infants in the both grips condition who activated their puzzle box took significantly longer to do so for the light box. Several potential explanations may explain this increased latency to success. First, the both grip condition included more modeling trials (four) than the other modeling conditions (three), potentially increasing cognitive load or fatigue. Second, attending to multiple grip types and modeling stimuli may have placed greater demands on infants' cognitive resources. Third, exposure to conflicting grip information may have made it more difficult to integrate the modeled actions with infants' existing knowledge about how tools should be grasped. For example, baseline grip data suggested that infants generally preferred to grasp the tool by the narrow end, but this grip was not compatible with activating the light box. Thus, exposure to both grip types (i.e., preferred narrow end grip and compatible wide end grip) may have led to uncertainty about which grip to use. Any combination of these factors could have contributed to the pattern of results we obtained.

Although we did not have a priori hypotheses about how long it would take to activate each puzzle box, we found that, on average, infants took longer to activate the sound box than the light box. Our interpretation of this result is that the kinematics of the light box (i.e., inserting the tool on the side of the box) were more straightforward than those of the sound box (i.e., inserting the tool on the top of the box). It may have taken longer for infants to lift the tool up over the sound box to insert the tool on the top of the box than to lift it slightly off the table to insert it into a hole on the side of the sound box. We also found no difference in how likely infants in the control condition were to activate the puzzle boxes overall. Taken together, these results suggest that one box was not inherently more difficult to activate than the other.

### 4.2 Generalization of Tool Use Knowledge

Our results demonstrate that infants transfer knowledge of tools beyond the specific information given to them in two ways. First, the modeling activity was different from the test activity. During the modeling phase, infants watched an adult use a novel, spatula‐like tool to push pom‐poms out of a tube (e.g., large pom‐poms and a wide tube for the narrow grip model; small pom‐poms and a narrow tube for the wide grip model). In contrast, test trials involved infants using the same or a similar tool to activate a puzzle box. Our finding that different modeling conditions in one context resulted in distinct patterns of performance in another suggests that infants were flexible enough to apply what they learned in one kind of task to another.

Secondly, we investigated whether infants would extend their learning from the red tool used during modeling to the green and white tools used in the second and third test trials. Because successfully activating each puzzle box required a specific grasp on the tool (light box: wide end grasp; sound box: narrow end grasp, see Figure 2), we considered success on each puzzle box as evidence of gripping the required end of the tool. Success rates were not significantly different for the green tool, which differed only in color from the red tool. In contrast, infants were less successful with the white tool, which differed from the red tool in shape, pattern, and color. Thus, the infants generalized what they learned about a new tool from a brief observation to a tool that differed in only one perceptual feature but had difficulty generalizing this knowledge to a more perceptually distinct tool with the same action‐relevant features.

This pattern of results is consistent with other findings in the literature on infants' object perception and representation that show more reliance on form features (e.g., shape) than on surface features like color and pattern. Studies by Needham (1999) showed that 4‐month‐old infants use changes in object shape but not changes in object color and pattern as indicators of an object boundary. Similarly, findings reported by Wilcox (1999) showed that, looking developmentally, younger infants are more likely to use a difference in object shape to individuate an object than they are to use a difference in object color or pattern. Thus, the current findings build on this earlier work showing that changes in object shape are regarded as more likely to signal fundamental differences in objects than are changes in color and pattern.

This difference could be related to how visual information is processed in the brain, because form features are closely linked to where an object is and how one would prepare to grasp the object, whereas differences in color are more closely linked to the identity of the object (De Yoe and Van Essen 1988; Goodale and Milner 1992). Extensive evidence now shows that the dorsal pathway for processing visual information in the brain focuses on where an object is and/or how one would act on it, whereas the ventral pathway is responsible for determining an object's identity (Goodale and Milner 1992; Mishkin and Ungerleider 1982). Furthermore, infants may be more likely to include objects of similar shape into the same category than they are to group objects of similar color and pattern into the same category (Landau et al. 1988; but see also Cimpian and Markman 2005). Thus, there are multiple reasons why infants would have extended what they learned about the red tool to the green tool, but not to the white tool.

### 4.3 Limitations and Future Directions

One notable limitation of the current study is that we tested only a small subset of tools with limited variation, and the variation was not done systematically (i.e., the last trial involved a tool which differed in both color, pattern, and shape instead of only varying in one dimension). Further, it is possible that differences in performance may have been in part due to varying levels of interest in the puzzle boxes themselves. However, when averaging across modeling groups, we did not observe any puzzle box‐level differences in solve likelihood. Further, although we did not directly test infant interest in each puzzle box, we did observe that the number of infants who solved the light box with a tool at baseline (*n* = 3) was equal to the number of infants who solved the sound box with a tool (*n* = 3).^1^ Together, these observations suggest that no box was significantly more motivating to solve than the other, although more direct testing of infant interest in the puzzle box outcomes would be needed to say conclusively either way. Future research should systematically manipulate the properties of tools and puzzle boxes to determine the specific factors (e.g., tool‐level properties, infant motivation, prior experience) that facilitate or limit knowledge transfer across tools.

In addition, we are aware that by testing infants' behaviors using experimenter‐designed tasks in a laboratory setting, we may end up overlooking key components of how infants really think about and use tools in their natural home environments. We are currently investigating these exciting questions about what infants' tool use looks like when they are playing at home in their natural environments (Danforth et al. manuscript submitted for publication). By combining insights from controlled, lab‐based studies with detailed observations of infants at home, we can develop a more nuanced understanding of how skills like tool use develop early in life.

Tool use is a remarkably multi‐faceted process. As we mentioned in the Introduction (and as others have written before us) tool use is necessarily dependent upon many factors, including the actor's motor abilities, cognitive skills, and even prior experiences due to cultural practices (Adolph and Hoch 2019; Creem and Proffitt 2001; Franchak and Adolph 2014; Kahrs et al. 2013; Legare 2019; Lockman 2000). Although this study's focus was on the cognitive influences of tool use and we obtained clear evidence to support these effects, this does not mean that cognitive factors are the only factors to consider when trying to understand the development of infants' tool use. We designed these tasks to be within the motor repertoires of typically developing infants in this age range, and we kept these demands as consistent as possible across tasks. We did not address effects of other possible influences on these tasks, but we created tasks that our participants would not have had prior experiences with.

## 5 Conclusions

The findings of this project show that infants in this age range can learn about a new grasping strategy for a tool based on a very brief modeling demonstration in one kind of task (pushing pom‐poms out of a tube) and use the tool with that same grip for a different task (solving a puzzle box). It becomes more challenging for them to extend what they have learned from one tool to another tool, however, as they succeed in activating a puzzle box using the same grasp strategy with a highly similar tool (differing only in color), but are not as successful with a less similar tool (differing in color, pattern, and shape). We created a situation in which infants had very little information to use to solve the challenges we gave them, as both the tools and tasks were unfamiliar. In such circumstances, these inexperienced tool‐users may rely more on the surface‐level perceptual features than they would if they had more abstract representations of tool categories and functional properties that experienced tool‐users may have access to.

This project focused on two foundational cognitive processes, observational learning and generalization, in the tool use of infants during the second year of life. It provides important insights into early tool use development by demonstrating that infants' emerging tool use abilities are the result of a complex interplay between what they observe from skilled tool users in their environment and perceptual constraints on their knowledge transfer. Overall, our results indicate that brief experience watching a tool being used influenced infants' subsequent tool‐using, and that the nature of infants' observational experience with tools and the characteristics of the tools themselves influenced infants' ability to generalize their learning to new tool‐using opportunities.

## Author Contributions

**Caroline Danforth:** writing – original draft, writing – review and editing, investigation, formal analysis, methodology, visualization. **Jane Hirtle:** conceptualization, investigation, methodology, formal analysis. **Supreethi Sridhar:** methodology, writing – review and editing, investigation, data curation. **Amy Work Needham:** funding acquisition, methodology, writing – review and editing, writing – original draft, supervision, project administration, conceptualization.

## Ethics Statement

All caregivers gave informed consent, and all aspects of the study were conducted in accordance with the ethical standards of the American Psychological Association. The data in the study were collected with approval from Vanderbilt University's institutional review board (IRB #: 090654).

## Conflicts of Interest

The authors declare no conflicts of interest.

## Data Availability

Data will be made available upon request.

## Endnotes

^1^
Infants who solved the task during the pre‐modeling baseline phase were not included in success or latency analyses.
